# Food Consumption According to the Days of the Week – National Food Survey, 2008-2009

**DOI:** 10.11606/S1518-8787.2017051006053

**Published:** 2017-11-07

**Authors:** Luana Silva Monteiro, Bruna Kulik Hassan, Camilla Chermont Prochnik Estima, Amanda de Moura Souza, Eliseu Verly, Rosely Sichieri, Rosangela Alves Pereira

**Affiliations:** I Curso de Nutrição. Universidade Federal do Rio de Janeiro. Macaé, RJ, Brasil; II Programa de Pós-Graduação em Saúde Coletiva. Instituto de Medicina Social. Universidade do Estado do Rio de Janeiro. Rio de Janeiro, RJ, Brasil; III Instituto de Estudos em Saúde Coletiva. Universidade Federal do Rio de Janeiro. Rio de Janeiro, RJ, Brasil; IV Departamento de Epidemiologia. Instituto de Medicina Social. Universidade do Estado do Rio de Janeiro. Rio de Janeiro, RJ, Brasil; V Departamento de Nutrição Social Aplicada. Instituto de Nutrição Josué de Castro. Universidade Federal do Rio de Janeiro. Rio de Janeiro, RJ, Brasil

**Keywords:** Food Consumption, Energy Intake, Feeding Behavior, Diet Surveys, Consumo de Alimentos, Ingestão de Energia, Comportamento Alimentar, Inquéritos sobre Dietas

## Abstract

**OBJECTIVE:**

Evaluate the variations in energy, nutrients, and food groups intake between days of the week and weekend days in the Brazilian population.

**METHODS:**

We used data from the first National Food Survey (2008-2009) of a one-day food log of a representative sample of the Brazilian population aged 10 years or older (n = 34,003). For the analyses, we considered the sample weights and the effect of the study design. The mean (and standard deviations) and frequencies (%) of energy, nutrients, and food groups consumption were estimated for weekdays (Monday to Friday) and weekend (Saturday and Sunday), we then estimated the differences according to the days of the week for the population strata analyzed.

**RESULTS:**

The average daily energy intake for the weekend was 8% higher than the one observed for weekdays. The average percentage contribution of carbohydrate to the daily energy intake was higher during the week compared to Saturday and Sunday (56.3% *versus* 54.1%, p < 0.01). The inverse was observed for averages of the contribution to the daily intake of energy from total fat (26.8% *versus* 28.4%), saturated fat (9.1% *versus* 9.9%) and trans fat (1.4% *versus* 1.6%). The most significant changes between weekdays and weekend days were observed for eggs, sugar-added beverages, puff snacks and chips, beans, and pasta. During weekends, the frequency of beverage with added sugar consumption increased by 34%, the amount consumed increased by 42%, and the contribution to energy intake increased by 62% when compared to weekdays.

**CONCLUSIONS:**

The Brazilian population increases energy intake and unhealthy food markers on weekends compared to weekdays.

## RESUMO

**OBJETIVO:**

Avaliar as variações na ingestão de energia, nutrientes e grupos de alimentos entre dias da semana e dias de fim de semana na população brasileira.

**MÉTODOS:**

Foram utilizados dados obtidos no primeiro Inquérito Nacional de Alimentação (2008-2009) de um dia de registro alimentar de amostra representativa da população brasileira com 10 ou mais anos de idade (n = 34.003). Para as análises, foram considerados os pesos amostrais e o efeito do desenho de estudo. Foram estimadas as médias (e desvios-padrões) e frequências (%) da ingestão de energia, nutrientes e grupos de alimentos para dias de semana (de segunda a sexta-feira) e de final de semana (sábado e domingo), sendo estimadas as diferenças segundo os dias da semana para os estratos populacionais analisados.

**RESULTADOS:**

A ingestão diária média de energia do fim de semana foi 8% maior que a observada para dias de semana. A contribuição percentual média de carboidrato para a ingestão diária de energia foi maior durante a semana em comparação com sábado e domingo (56,3% *versus* 54,1%; p < 0,01). O inverso foi observado para as médias da contribuição para a ingestão diária de energia de gordura total (26,8% *versus* 28,4%), gordura saturada (9,1% *versus* 9,9%) e gordura trans (1,4% *versus* 1,6%). As mudanças mais importantes entre os dias de semana e de fim de semana foram observadas para ovos, bebidas com adição de açúcar, salgadinhos e *chips*, feijão e massas. Nos finais de semana, a frequência de consumo de bebidas com adição de açúcar aumentou em 34%, a quantidade consumida sofreu incremento de 42% e a contribuição para a ingestão de energia se elevou em 62%, quando comparados aos dias de semana.

**CONCLUSÕES:**

A população brasileira aumenta a ingestão de energia e de marcadores de alimentação pouco saudável nos finais de semana em comparação aos dias de semana.

## INTRODUCTION

In the last few decades, significant changes occurred in food consumption in several countries^1^. In Brazil, there has been an increase in the participation of processed products in the availability of food in Brazilian households^18^. Data from Family Budget Surveys showed that, between 2002-2003 and 2008-2009, the relative participation of cookies, soft drinks and ready meals in the availability of energy at home increased by 10%, 16%, and 40%, respectively^16^. The process of eating in Brazil has been set up as being of high energy density, high content of sugars and sodium and low content of fibers and micronutrients. Thus, our diet is an important risk factor for increasing the prevalence of obesity and chronic non-transmissible diseases^20^ ^,^ ^25^.

It should be emphasized that the days of the week are classically considered as sources of intraindividual variability of food consumption^2^, considered important in the evaluation of error in food and nutrient consumption estimates in food surveys based on a 24-hour recall and food diary survey^29^.

Studies that evaluated food consumption according to the days of the week reported that food consumption during weekends presented worse nutritional quality than on weekdays, due to higher consumption of soft drinks and other sugary drinks, alcoholic drinks and fats, and lower participation of whole foods, leading to higher energy intake during weekends^10^ ^,^ ^23^ ^,^ ^24^.

In addition, the systematic increase in energy intake can lead to a positive energy balance and favors weight gain, as shown by Brown et al.^4^ These authors, when evaluating middle-aged women included in the Australian Longitudinal Study on Women’s Health, observed that an energy imbalance of only 10 kcal per day was associated with an average weight gain of approximately 0.5 kg per year^4^.

There are few studies evaluating changes in eating habits according to the days of the week, particularly in Brazil. The characterization of the food consumption according to the days of the week can contribute to the refinement of the food consumption evaluation and support actions of health promotion and healthy eating. The present study aimed to estimate the variations in energy, nutrients, and food groups intake between days of the week and weekend days in the Brazilian population.

## METHODS

Data from the first National Food Survey (INA) were used, included as a module of the Family Budget Survey (POF 2008-2009), which was developed by the Brazilian Institute of Geography and Statistics (IBGE). The INA was performed in a subsample of the households included in the POF 2008-2009 and a total of 34,003 residents who were least 10 years old participated in the survey^11^.

Food consumption was estimated using two non-consecutive dietary records obtained in one week. Participants were instructed to report all food and beverages (except water) consumed, the amount consumed, the day of the week, and the time and place of consumption (inside or outside the home). This study used data from the food registered on the first day by the evaluated population.

Intake of energy, carbohydrate, lipid and protein, saturated fat, trans fat, and added sugar was estimated based on the composition table of foods consumed in Brazil^13^.

The foods cited in the food records were categorized into 58 groups, according to the grouping adopted by Pereira et al.^21^ Briefly, this grouping system considers nine main groups disaggregated into subgroups according to the nutritional characteristics of foods and beverages and their use in the diet. Many subgroups considered in this categorization were reported in very low proportions in the INA (< 2%). For the analysis of the variation in the prevalence of consumption between weekdays and weekends, we decided to evaluate only the subgroups of foods referred by at least 10% of the population (20 subgroups) (Box).

**Box:** Food groups cited by the Brazilian population. National Food Survey (INA).

Food groups	Food cited
Eggs	Chicken egg, quail egg, and omelet
Sugar-added beverages	Soft drinks, guarana syrup, soda, and powdered juices
*Puff snacks and chips*	Salty bagel, salty cracker, chips, sweet or salty popcorn
Bean	Beans, lentils, chickpeas, soybeans
Pastas	Macaroni, lasagne, capeletti, cannelloni, gnocchi, ravioli, yakissoba
Fruit juice	Pineapple juice, acerola juice, cupuaçu juice, guava juice, orange juice, orange juice with banana, papaya juice, mango juice, passion fruit juice, strawberry juice, peach juice, organic juice
Oil and fat	Butter or margarine with salt or without salt, bottle butter, pork rind, pork crackling (*pururuca*), olive oil
Vegetables	Pumpkin, zucchini, chard, watercress, lettuce, garlic, leek, common chicory, asparagus, eggplant, Malabar spinach, beetroot, broccoli, alfalfa sprouts, bean sprouts, onion, chives, carrots, chicory, cauliflower, pea pod, escarole, spinach, palm heart, scarlet eggplant, cucumber, bell pepper, okra, radish, cabbage, arugula, taro, tomato, green bean, roselle
Fruits	Avocado, pineapple, abiu, acai berry, acerola, plum, blackberry, atemoya, bacuri, banana, biriba, ambarella, cashew, sugar cane, persimmon, starfruit, cherry, jocote, cupuaçu, fig, sweetsops, guava, soursop, imbu, inga, jabuticaba berry, jackfruit, jambrosade, jenipapo, jurubeba, kiwi, lemon, apple, papaya, mango, mangaba, passion fruit, watermelon, melon, tangerine, nache, strawberry, raisins, pequi, pear, peach, Brazilian cherry, pitomba, pomegranate, sapodilla, tanja, umbu, grape
Cheese	Danbo-type cheese, colony cheese, muzzarella cheese, kingdom cheese, minas cheese, curd cheese, canastra cheese, ricotta cheese, polenguinho cheese, grated cheese, gorgonzola cheese, cheese spread, unspecified cheese
Processed meats	Ham-like meat, turkey blaquet, Sun dried meat, dried meat, chorizo, sausage, mortadella, corned beef, paté, turkey breast, ham, salami, sausage, tender
Poultry and poultry preparations	Chicken, chester, quail, chicken gizzard, chicken liver, chicken burger, duck, turkey
Breads	Bread with butter/margarine, hamburger bread, loaf of bread, salt bread, small dinner roll, whole-wheat bread, unspecified bread, toast of any bread
Rice	Rice (polished, parboiled, needle, *agulhinha*, etc.), rice starch, greek rice, *arroz carreteiro*, rice and beans, rice and cassava, rice and eggs, rice and milk, brown rice, oat flakes, rice cream, porridge (rice, cornmeal, oats, flour, etc.), risotto
Meat and meat preparations	Beef, goat, caprine, lowland paca, goat, goatling, capybara, sheep, alligator, pork
Sweets and desserts	Alfajor biscuit, sweet cookie, sandwich cookie, cakes, brevidade cake, panettone, churros, cuca cake, *cavaca*, *filhós*, honey bread, sweet bread pave, french toast, swiss roll, sweet donut, *quebra-queixo*, *sequilho*, berliner, pies
Coffee and tea	Traditional mate, mate herb, *chimarrão*, tea, organic mate, tea with flour, instant coffee, cappuccino, coffee, latte
Candies and chocolates	Chocolate peanut, candies, *beijo de moça*, chocolate bar, bonbon, brigadeiro, stuffed straw, chewing gum, cocada, egg-based sweet, peanut candy, sweet candied fruit, fruit candy, ice cream, guava, jelly beans, maria mole, meringue, peanut candy, *pé de moleque*, popsicle, lollipop, torrone, swiss meringue
Roots and tubers	Cassava, potato, sweet potato, carrot, yam, arracacha, corn, tapioca
Milk	Whole cow’s milk, fresh cow’s milk, goat’s milk, whole milk powder, skimmed milk powder, milk powder, fermented milk, flavored milk, milk with chocolate, skimmed cow’s milk, semi-skimmed cow’s milk, unspecified pasteurized milk

Reports between Monday and Friday were considered as weekdays, and Saturdays and Sundays were treated as weekend consumption (Weekend). The inclusion of Friday on weekdays was based on exploratory analyses that allowed us to identify that, in the studied population, the consumption of energy and macronutrients for Friday was more compatible with consumption from Monday to Thursday than from Saturday to Sunday. The data were evaluated by gender, age group, and by *per capita* family income. In the categorization by age group, we considered adolescents between 10 and 19 years old, adults between 20 and 59 years old, and older adults were those aged 60 and over.

To estimate the *per capita* family income, the total household income was divided by the number of residents. Family income was categorized into five groups: < 0.5 minimum wage *per capita*, 0.5 ≤ minimum wage *per capita* < 1, 1 ≤ minimum wages *per capita* < 2, 2 ≤ minimum wages *per capita* < 5 and ≥ 5 minimum wages *per capita*. For this classification, the value of the minimum wage was R$415.00 (four hundred and fifteen reais) in use on January 15, 2009, the reference date of the survey^12^.

The data were analyzed using the Statistical Package for Social Sciences (SPSS), version 19. In the analyses, we considered the sample weights and the effect of the sample design.

Variations in energy and nutrient intake among the population strata were estimated using generalized linear models (GLM) developed in the Complex Sample module, with correction for Bonferroni in the comparison according to income class. We evaluated the differences between the population strata of the categorical variables through the chi-squared test.

The research protocol was approved by the Ethics Committee of the Instituto de Medicina Social of the Universidade do Estado do Rio de Janeiro (CAAE 0011.0.259.000-11, of July 19, 2011).

## RESULTS

Only subjects aged ≥ 60 years old did not change their dietary intake between weekdays and the weekend for any of the components analyzed. In adults, changes were observed for all dietary factors evaluated. Among adolescents, there was a reduction in the contribution of carbohydrates to energy intake and an increase in the participation of total lipids and trans fat in daily energy intake. For women, only added sugar did not show any variations. For men, besides added sugar, there was also no variation for carbohydrates.

There was a mean increase of 8% in the daily energy intake in weekends when compared to weekdays (2,059 kcal *versus* 1,906 kcal; p < 0.01). Significant differences in daily energy intake between weekdays and weekends were observed for women and men, adults and individuals with *per capita* income above two monthly minimum wages. This difference was more significant among the individuals who belonged to families with monthly income ≥ 5 minimum wages *per capita*, for which a mean increase of 16% in daily energy intake was observed, which was 2.3 times higher than the one observed in the general population (Table 1).

**Table 1:** Energy intake and contribution (%) of macronutrients to daily energy intake on weekdays and weekends according to socioeconomic characteristics. National Food Survey, Brazil, 2008–2009.

Group	Energy		Carbohydrates		Total lipids		Saturated fat		Trans fat		Sugar-added	
					
	(kcal)		(%)		(%)		(%)		(%)		(%)	
					
	Week	Weekend	Week	Weekend	Week	Weekend	Week	Weekend	Week	Weekend	Week	Weekend
Brazil (total)	1,906^a^	2,059	56^a^	54	27^a^	28	9.2^a^	10.0	1.4^a^	1.6	13^a^	14
Week-Weekend variation (%)	7		-4		6		8		13		4	
Gender												
Female	1,710^b^	1,879	57^b^	55	27^a^	29	9.3^b^	10.1	1.4^b^	1.6	14	15
Week-Weekend variation (%)	9		-4		7		8		13		5	
Male	2,117^b^	2,250	56	54	27^b^	28	9.0^b^	9.8	1.3^a^	1.5	13	13
Week-Weekend variation (%)	6		-4		4		8		13		0	
Age group												
Adolescent (10–19 years old)	2,058	2,055	57^a^	55	27^b^	29	9.3	10.3	1.5^a^	1.7	15	15
Week-Weekend variation (%)	0		-4		8		10		12		0	
Adult (20–59 years old)	1,913^b^	2,107	56^b^	54	27^b^	28	9.1^b^	9.9	1.3^b^	1.5	13^a^	14
Week-Weekend variation (%)	9		-4		3		8		13		5	
Older adults (≥ 60 years)	1,633	1,727	56	54	27	28	9.2	9.6	1.3	1.4	11	11
Week-Weekend variation (%)	5		-4		5		4		7		0	
Family income *per capita* (minimum wage)												
< 0.5	1,791	1,800	58^a^	56	25^b^	28	7.9^b^	9.1	1.2^b^	1.7	12	13
Week-Weekend variation (%)	1		-4		10		13		29		4	
0.5–1	1,901	2,069	57^a^	54	26	28	8.8	9.6	1.4^a^	1.6	13	13
Week *versus* Weekend variation (%)	8		-5		5		8		13		0	
1–2	1,927	2,011	56^b^	54	27	28	9.3	9.6	1.4	1.5	13	13
Week *versus* Weekend variation (%)	4		-5		3		3		7		0	
2–5	1,975^b^	2,018	55^a^	54	28^b^	30	9.9^a^	10.7	1.5	1.4	14^a^	15
Week-Weekend variation (%)	2		-2		5		7		7		9	
≥ 5	1,917^b^	2,277	54	53	28	29	10.6	11.4	1.2	1.3	13^a^	15
Week-Weekend variation (%)	16		-2		3		7		8		16	

Week: weekdays (from Monday to Friday); Weekend: weekend days (Saturday and Sunday)

% Variation: (Week-Weekend)/Week*100

^a^ p < 0,05

^b^ p < 0,01

The percentage contribution of carbohydrates to daily energy intake was lower in the weekends, and this reduction was, on average, of 4%, with no significant oscillations observed between the different strata. However, for men, older adults, and individuals with monthly income ≥ 5 minimum wages *per capita*, there was no change in the proportion of daily energy provided by carbohydrates (Table 1).

The percentage contribution of total lipids, saturated fat, and trans fat in the weekends was higher than on weekdays. These changes occurred similarly in all strata analyzed (sex, age range, and income). The most significant increases in the contribution of total lipids, saturated fat, and trans fat to daily energy intake were observed for the monthly family income range below 0.5 minimum wage *per capita*. In this group, the contribution of total lipids to daily energy increased, on average, 10% between weekdays and weekends, from 25% to 28% (p < 0.01); saturated fat, during the days of the week corresponded, on average, to 7.9% of the daily energy intake and on weekends, to 9.1%, which represented an average increase of 13% (p < 0.01); for trans fat, the mean increase over the days of the week was 29% (p < 0.01) and this rate was higher than twice the average increase observed in the general population (Table 1).

We observed that the contribution of added sugar to the daily energy intake increased over the weekend in relation to the weekdays and the most significant increases occurred among individuals from families with monthly income *per capita* ≥ 5 minimum wages. In this group, the participation of added sugar in daily energy intake between weekdays and weekends increased from an average of 13% to an average value of 15%, which represented an average increment of 16% (p < 0.05). This increase was four times higher than the mean increase in the general population (Table 1).

The most consumed foods and those that represented the highest caloric contribution to daily energy intake both on weekdays and on the weekends, were rice, coffee or tea, beans, bread, and meats. Food groups whose prevalence of consumption varied significantly (> 20% variation) between the weekdays and weekends were: eggs (-35%), sugar-added beverages (+34%), puff snacks and chips (-22%), beans (-21%), and pasta (+20%) (Table 2).

**Table 2:** Comparison between the consumption of food groups between weekdays (Week) and weekend days (Weekend). National Food Survey, Brazil, 2008–2009.

Group	Prevalence of consumption			Quantity consumed			Contribution to daily energy intake^a^		
		
	Week	Weekend	Variation Week-Weekend	Week	Weekend	Variation Week-Weekend	Week	Weekend	Variation Week-Weekend
	%	%	%	grams	grams	%	%	%	%
Eggs	17^b^	12^b^	-35	9.2^b^	6.6^b^	-39	1.5^c^	1.0^c^	-33
Sugar-added beverages	29^b^	44^b^	34	101.7^b^	174.5^b^	42	2.1^c^	3.4^c^	62
Puff snacks and chips	19^c^	16^c^	-22	4.3^b^	4.6^b^	7	1.9^b^	1.5^b^	-21
Bean	78^b^	65^b^	-21	134.7^b^	103.6^b^	-30	10.5^c^	8.1^c^	-23
Pastas	21^c^	26^c^	20	28.9^c^	41.2^c^	30	3.3^b^	4.3^b^	30
Fruit juice	34^c^	29^c^	-18	109.6^c^	92.2^c^	-19	4.8^c^	3.8^c^	-21
Oil and fat	37^b^	43^b^	14	2.5^c^	2.9^c^	14	2.3^b^	2.7^b^	17
Vegetables	43^c^	37^c^	-14	27.6^b^	24.9^b^	-11	0.6^b^	0.5^b^	-11
Fruits	35^b^	31^b^	-13	66.3^b^	59.0^b^	-12	3.1^b^	2.6^b^	-16
Cheeses and cheese products	14	15	11	4.1	4.8	15	1.0	1.1	10
Processed meat and processed meat preparations	19	21	9	6.3	5.9	-7	1.9	1.7	-11
Poultry and poultry preparations	27	29	7	22.8	26.8	15	4.8	4.7	-2
Bread	64	67	5	38.6	40.4	4	8.8	8.9	1
Rice	91^c^	88^c^	-4	91.4^b^	80.4^b^	-14	14.8^c^	13.1^c^	-11
Meat and meat preparations	53	55	4	47.5	50.8	6	10.0	10.7	7
Sweets and desserts	28	27	3	16.7^b^	20.6^b^	19	4.9	5.2	6
Coffee and tea	82	81	1	233.7	227.7	-3	6.5	6.3	-3
Candies and chocolates	12	12	0	6.2	6.7	7	1.1	1.2	12
Roots and tubers	27	27	0	19.3	18.1	-7	3.8	4.0	5
Milk	23	23	0	57.9	63.6	9	2.4	2.5	4

Week: weekdays (from Monday to Friday); Weekend: weekend days (Saturday and Sunday)

^a^ Contribution to daily energy intake - (food group energy*100)/total energy intake.

^b^ p < 0,05

^c^ p < 0,01

For men, on weekdays and weekends, we observed a reduction in the prevalence of rice and beans consumption and an increase in the prevalence of consumption of oils and fats, eggs, and beer. For women, there was a reduction in the prevalence of consumption of beans, vegetables, fruit juices, and puff snacks and chips. For both men and women, the prevalence of sugar-added beverages and pasta increased in the weekends compared to the weekdays. The prevalence of bean consumption was reduced for all age groups. Among adults, in weekdays and weekends, the prevalence of consumption of oils and fats, pasta, and beer increased; on the other hand, the prevalence of consumption of rice, vegetables, eggs, and maize and corn preparations decreased. During the days of the week and weekends, adolescents and adults also reduced the prevalence of consumption of fruit juices and puff snacks and chips, and older adults only increased the frequency of consumption of oils and fats. We found that beer was among the 20 foods most consumed by adults and men during weekends and that their consumption in the weekends was three times higher than that estimated for weekdays (data not shown).

Comparing dietary intake between weekdays and weekends, changes were observed in the caloric contribution of food to daily energy intake. The most relevant changes (> 20%) were observed for sugar-added beverages (+62%), eggs (-33%), pasta (+30%), beans (-23%), fruit juices (-21%), and puff snacks and chips (-21%). In addition, there was also a reduction in the caloric contribution of fruits, vegetables, and rice (Table 2). For eggs, beans, fruit juices, vegetables, fruits, and rice, there was a reduction both in the prevalence of consumption and in the quantities consumed, as well as in the caloric contribution between the weekdays and weekends, with the most important reductions observed for eggs and beans. On the other hand, during weekdays and weekends, for sugar-added beverages, pasta, and oils and fats, there was an increase both in the prevalence of consumption and in the quantities consumed and in the contribution to energy intake. The significant variation observed for sugar-added beverages, with a 42% increase in the amounts consumed and 62% of the contribution to the daily energy intake (Table 2), should be noted.

During weekdays and weekends, there was a reduction in the average amounts consumed of eggs, beans, fruit juices, rice, fruits, and vegetables; on the other hand, there was an increase in the average amounts consumed of sugar-added beverages, pasta, sweets and desserts based on flour, oils and fats, and puff snacks and chips. It should be noted that the most significant variations (> 20% variation) in the amounts consumed were observed for sugar-added beverages (+42%), eggs (-39%), beans (-30%), and pasta (+30%) (Table 2).

**Figure 1:**
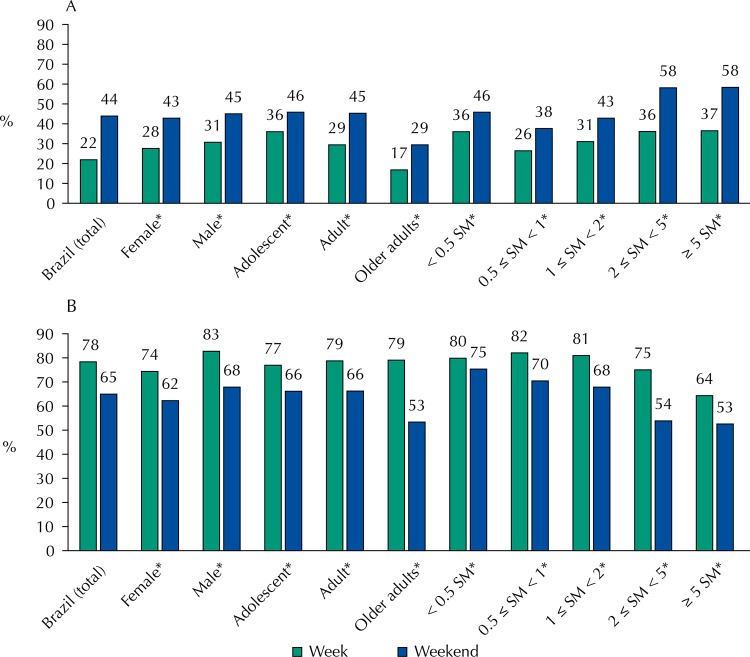
Prevalence of sugar-added beverages (A) and beans (B) on weekdays and weekends. National Food Survey, Brazil, 2008–2009.

**Figure 2:**
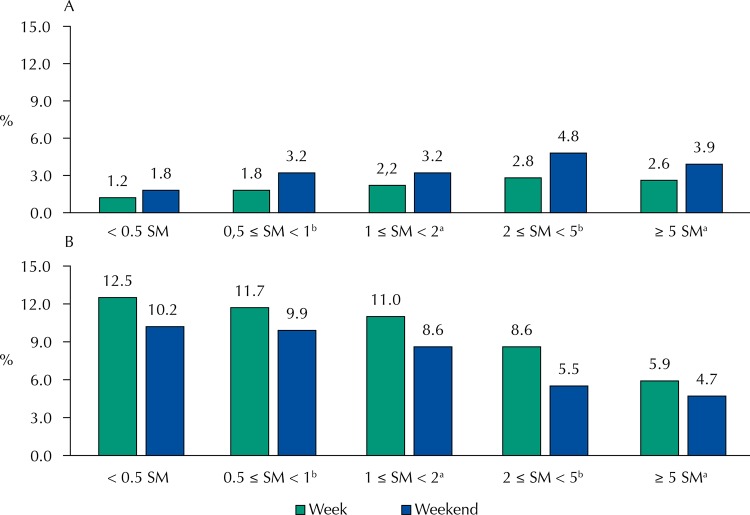
Contribution (%) of sugar-added beverages (A) and beans (B) for daily energy intake, on weekdays and on weekends according to income. National Food Survey, Brazil, 2008–2009.

Figures 1 and 2 show the variations between weekdays and weekends recorded for sugar-added beverages and beans, as these groups presented the most significant variations and underwent significant modifications for almost all categories of variables analyzed. Compared with weekdays, the statistically significant increase in the frequency of consumption of sugar-added beverages in weekends was observed in all strata analyzed, except for older adults. For beans, the reduction in the frequency of consumption between weekdays and weekends was also consistent for all strata evaluated, except for individuals belonging to families with monthly income < 0.5 minimum wage *per capita* (Figure 1, A).

SM: minimum wage

* p < 0,01

SM: minimum wage

^a^ p < 0,05

^b^ p < 0,01

We observed variations between the days of week and weekends in the caloric contribution for the daily energy intake of sugar-added beverages and beans. For example, there was a reduction in the average caloric contribution of beans (10.5 *versus* 89%; p < 0.01), except among individuals with monthly income between 1 e 2 and ≥ 5 minimum wages *per capita* (Figure 2, B). For sugar-added beverages, the caloric contribution did not increase for individuals with monthly income < 0.5 minimum wages *per capita* (Figure 2, A).

## DISCUSSION

This is the first study that compared the intake of energy, nutrients, and food between weekdays and weekends in a representative sample of the Brazilian population. This study showed that during weekends significant changes in food consumption occur in comparison to the days of the week. During weekends, there was a higher consumption of energy, lipids, saturated and trans fats, and added sugar. These findings may be related to the reduction, during weekends, of the frequency and quantities of food groups consumed that are part of the traditional pattern of Brazilian food and healthy food markers (rice, beans, fruits, and vegetables), together with the increase in consumption of foods such as sugar-added beverages, oils and fats, and pasta.

Surveys conducted in the United States and Finland^5^ ^,^ ^10^ ^,^ ^14^ have observed results similar to those of the present study, particularly with regards to increased energy intake during weekends in relation to days of the week. Haines et al.^10^, identified higher energy consumption during weekends (from Friday to Sunday) for the total sample evaluated, with a more significant increase in the age group between 19 to 50 years. In addition, they found an increase in the consumption of fat and alcohol for adults and a decrease in the proportion of carbohydrate ingested during weekends compared to the weekdays. Haines et al.^10^ and Jula et al.^14^ considered weekends as the period between Friday and Sunday. In the present study, the period from Saturday to Sunday was used, since the consumption of energy and macronutrients referring to Friday by the population studied was similar to consumption from Monday to Thursday.

The Brazilian population’s increase in caloric intake during the weekend was on average 153 kcal, which can lead to an average increment of 1,224 kcal over a month and almost 15,000 kcal at the end of a year. This increase, if not offset by increased energy expenditure, can lead to a gain of 2 kg in a year. Thus, this variation in energy intake according to the weekdays may be a factor contributing to the increase in the prevalence of overweight and obesity in the country^12^ ^,^ ^19^. Other studies related the increase in caloric intake during weekends with the increase in body weight^7^ ^,^ ^22^. Craemer et al.^7^, in order to evaluate whether changes in diet or physical activity during the week contributed to weight gain or to impede weight loss, developed a systematic review with preschoolers and observed increases in weight gain during weekends but not on weekdays. This increase in weight was attributed to higher energy intake and reduction of physical activity practice at the weekend in relation to the days of the week^7^.

For Castro^5^, the observed differences in dietary intake between weekdays and weekends can be attributed to changes in the daily routine. During the week, mealtime is generally reduced due to work, study, and other duties^5^. During weekends, it is possible that there is more consumption outside the home, a higher frequency of celebrations and parties, and more time available for meals, conditions that may contribute to the increase of consumption during the weekend^5^ ^,^ ^6^ ^,^ ^8^.

The results of this study are consistent with the analyses that evaluated food consumption and food availability in the country. Pereira et al.^21^ evaluated data from this same survey and identified a high participation of foods with excessive content of solid fats and added sugar in the diet of Brazilians. Levy et al.^16^, when evaluating the household availability of food between 1987 and 2003, found that household availability of added sugar in Brazil reached 16.7% of the total calories available in households and that participation was high in all the regional and income strata.

Additionally, the results presented are also compatible with studies that evaluated food consumption by income class, which have observed that the higher income classes present more significant consumption of energy-rich foods, such as soft drinks^18^ ^,^ ^27^. In this analysis, individuals with a higher income (≥ 5 minimum wages *per capita*) presented a significant increase in the consumption of sugar-added beverages during weekends, which did not occur among individuals of the lowest income level (< 0.5 minimum wages *per capita*), although there has been an increase in the contribution of fats to daily energy intake.

Analysis based on a single day of food registration may be considered a limitation of this study. However, data from a single food log day obtained in a representative sample of the population can provide reliable estimates of population averages^9^. In addition, the quality of information on the first day has been considered superior to that recorded on subsequent days^28^. We chose to use only the first day of food registration because, when analyzing food, the proportion of zeros in the response is very high, which prevents adequate statistical adjustment^15^ ^,^ ^26^. In addition, since the dietary variable is dependent on the model, the effect of random error (intrapersonal variability) increases the total variance but does not influence the differences between the means. The variance increase could lead to the reduction of the sample’s power^3^; however, the sample size evaluated allows us to detect differences in the proportions of consumption of food groups and in the averages of energy, nutrients, and food groups intake with high power and level of confidence.

As a strong point of this study, we highlight the representativeness of the sample at the national level. Another aspect that can be considered a strong point of this study is the validity of the method applied to obtain food consumption data, evaluated in a study that adopted, as a reference for comparison, the estimate of the energy expenditure by doubly marked water, which indicated that the sub-report on energy intake was approximately 30%^17^.

This study found that, in Brazil, there is an increase in energy intake and markers of a diet related to deleterious effects on health during the weekends, especially sugar-added beverages. At the same time, on the weekends, there is a reduction in the consumption of healthy food markers, such as the combination of rice and beans, vegetables, and fruits. These findings should be the subject of studies that consider the repercussion of these changes over time since the systematic increase in energy intake can lead to an energy imbalance and favor excessive weight gain. Also, regular increases in the consumption of added sugar contained in beverages may increase the risk of developing metabolic disorders, such as insulin resistance and diabetes. The evidence observed in the present study should be considered in the formulation of initiatives to promote healthy eating, and the importance of maintaining healthy eating habits and physical activity on weekends should also be emphasized.
